# Effects of Nano-Aerators on Microbial Communities and Functions in the Water, Sediment, and Shrimp Intestine in *Litopenaeus vannamei* Aquaculture Ponds

**DOI:** 10.3390/microorganisms10071302

**Published:** 2022-06-27

**Authors:** Yingkai Xu, Lisong Li, Suo Lou, Jiashen Tian, Shuhao Sun, Xiaodong Li, Yingdong Li

**Affiliations:** 1 College of Animal Science and Veterinary Medicine, Shenyang Agricultural University, Dongling Road 120, Shenyang 110866, China; xuyingkai@syau.edu.cn (Y.X.); lisongli@syau.edu.cn (L.L.); slou@syau.edu.cn (S.L.); xiaodongli@syau.edu.cn (X.L.); 2 Liaoning Ocean and Fisheries Science Research Institute, Heishijiao Street 50, Dalian 116023, China; tianjiashen@163.com; 3 Dandong Fisheries Development Service Center, Zhongxin North Road 2, Dandong 118017, China; ddscssh@126.com

**Keywords:** *Litopenaeus vannamei*, nano-aerator, bacteria, intestine, water and sediment

## Abstract

Nanobubble technology has promising development and application prospects in the fields of sewage treatment, soil and groundwater remediation, animal and plant growth, and biomedicine. However, few studies have investigated its effect on shrimp aquaculture. In this study, we investigated the effect of nano-aerators on microbial communities of the water, sediment, and shrimp gut in a *Litopenaeus vannamei* aquaculture pond using 16S rRNA high-throughput sequencing. The results indicated that the nano-aerator significantly increased the microbial community diversity and species abundance in the pond, and the microbial community diversity of the pond sediment increased under short-term aeration conditions. Compared to that with ordinary aerators, nano-aerators increased the proportion of beneficial bacteria, such as *Exiguobacterium* and *Acinetobacter*, in the water and sediment microbial communities. Moreover, the proportions of beneficial bacteria in the gut, including *Rhodobacter*, *Oscillospira*, and *Faecalibacterium*, were all increased by using the nano-aerator. Therefore, our findings suggest that nano-aerators could promote the activity of beneficial bacteria in aquaculture ecosystems, thereby regulating water quality, reducing disease incidence, and improving aquaculture efficiency and benefits. Our findings provide new insights into the effects of nano-aerators on microbes in crustacean culture ponds.

## 1. Introduction

The dissolved oxygen in natural water sources is not sufficient to support high-stocking density aquaculture; therefore, artificial aeration has been increasingly used to increase the oxygen supply [1]. Mechanical aeration has become standard practice to prevent low levels of dissolved oxygen in fed feed-based farming models [1]. Currently, the types of aerators commonly used in pond culture are mainly impeller, waterwheel, and paddle wheel aerators [2]. Studies have shown that the impeller aerator has the highest aeration efficiency among the commonly used aerators at 2.098 kg O^2^/kW·h [3]. However, it must generally be fixed at one point in the pond, the aeration area is limited to a certain range, and the aeration effect is poor in larger aquaculture ponds [3]. Compared with other aerators, the advantage of the waterwheel aerator is that by turning the water body and pushing, making it flow, the water with high levels of dissolved oxygen is transported to the food court with water containing low-levels of dissolved oxygen, which greatly improves the feeding situation of the fish [4]. Studies have shown that it is more economical to use paddle wheel aerators in large ponds with volumes >5000 m^3^ [5]. However, with a substantial increase in the aquaculture density [6] and because some high-quality aquatic products have increasingly higher requirements for water quality, it is difficult for traditional aerators to aerate the water efficiently. The oxygen efficiency of traditional aerators is low, which restricts the further improvement of aquaculture production. Therefore, it is of great significance to test and promote efficient oxygenation technology, improve pond water quality, reduce disease occurrence, and further increase aquaculture production.

Recently, micro-nanobubble (MNB) technology has attracted much attention in many fields, such as surface water restoration [7], medicine [8], food science [9], and agriculture [10], owing to its special characteristics, such as a large oxygen-enhancing surface area. Studies have shown that MNB technology can improve the composition of microbial communities in water bodies to a certain extent [11] and subsequently restore water bodies; however, few studies have applied nanobubble technology to aquaculture production.

Probiotics are microbial products commonly used in aquaculture production, which can be used as feed additives [12] and amend water quality [13]. With the increase in research on probiotics, an expanded understanding of this concept has been achieved. Early work suggested that probiotics adapt specifically to life in animal intestines, which is beneficial to the health of the host microbes. Later studies have found that such microorganisms also exist in the environment, and they can change the environment to make it more suitable for animal life. Currently, probiotics or potential probiotics used in aquaculture mainly include species of *Actinomyces* [14], Bacteroidetes [15], Firmicutes [16], Proteobacteria [16], and yeast [17].

Presently, shrimp farming mostly utilizes high-density farming methods, and oxygen consumption is a considerable factor. When the dissolved oxygen content in water is <2.5 mg/L, the survival rate of *Penaeus chinensis* is substantially affected. Furthermore, periodic severe/moderate hypoxia can reduce the survival rate and growth performance of *Litopenaeus vannamei* [18]. Under hypoxic conditions, anaerobic microorganisms in the water environment will also become active and anaerobically ferment organic matter, producing many fermentation intermediates, such as hydrogen sulfide, methane, and ammonia, which can be toxic to farmed animals [19]. The accumulation of organic matter in the middle and late stages of high-density farming often leads to unstable water quality. Moreover, a large amount of organic matter will reduce the types of probiotics, reduce the stability of the microbial community in the water environment, and negatively affect aquaculture [20]. At the same time, shrimp ingest pathogens when they are markedly increased in the environment, thereby destroying the microbial community in the shrimp gut [21,22]. Studies have shown that the dissolved oxygen content significantly affects the release of phosphorus in sediment, and the release of phosphorus under anaerobic conditions is significantly higher than that under aerobic conditions [23]. However, an increase in the dissolved oxygen content in water will have a certain inhibitory effect on these anaerobic microorganisms, which is helpful to create a suitable breeding environment [24]. In a previous study, Kurita et al. [25] developed a new method for eradicating small planktonic crustaceans using “cavitation” shock waves based on MNB technology. However, there are few studies on the effects of MNB technology on water and sediment microbiota and environmental factors in shrimp ponds. Our goal was to examine these changes by high-throughput sequencing, followed by community diversity, species richness, and bioinformatic pathway analyses. This information is important for developing ecological strategies to prevent increases in pathogenic bacteria in the shrimp aquaculture environment [26].

## 2. Materials and Methods

### 2.1. Field Description and Experimental Design

The experiment was conducted over 14 weeks at Guanghe Fisheries Co. Ltd., Panjin City, Liaoning Province, China. Two adjacent *Litopenaeus vannamei* ponds were used as control and experimental pond groups, each with an area of 500 m^2^ (depth, 0.8 m). The experimental group was fitted with a nanobubble aerator with a power of 1 kW, and the control group was fitted with a common turbo-aerator with a power of 1 kW. Each pond was stocked with 40 prawns/m^2^, and the prawns were fed commercial feed (Wellhope Aquatic Feed Co. Ltd., Shenyang, China).

### 2.2. Sample Collection

The water and soil samples were collected on 8 August and 2 October 2021, respectively. Samples were collected at a depth of 0.5 m in the middle of the water body, and 100 mL water samples from five points in the same pond were equally mixed into one sample (500 mL in total). The topsoil was collected at a soil depth of 0–5.0 cm with the same method, and the soil samples collected at five points in the same pond were equally mixed into one sample, and three replicates were used, such that there were three soil samples in total. On 2 October 2021, the intestines of three shrimps (14.36 ± 1.65 g) from each pond were pooled into one sample, with quintuplicate samples per treatment. The collected samples were quickly frozen in liquid nitrogen and sent to Shanghai Personal Biotechnology Co., Ltd. (Shanghai, China) for DNA extraction and Illumina sequencing.

### 2.3. DNA Extraction

Intestinal genomic DNA samples were extracted using the OMEGA Soil DNA Kit (D5625–01; Omega Bio-Tek, Norcross, GA, USA) following the manufacturer’s instructions. A NanoDrop ND-1000 spectrophotometer (Thermo Fisher Scientific, Waltham, MA, USA) and agarose gel electrophoresis were used to measure the quantity and quality of the extracted DNA, respectively.

### 2.4. 16S rRNA Gene Amplicon Sequencing

The V3-V4 region of the bacterial 16S rRNA gene was PCR amplified with the forward primer 799F (5′-ACTCCTACGGGAGGCAGCA-3′) and reverse primer 1193R (5′-TCGGACTACHVGGGTWTCTAAT-3′). The PCR component contained 1 μL of DNA template. Thermal cycling conditions were as follows: initial denaturation at 98 °C for 5 min, followed by 25 denaturation cycles at 98 °C for 30 s, annealing at 53 °C for 30 s, and extension at 72 °C for 45 s, with a final extension at 72 °C for 5 min, using an Applied Biosystems 2720 thermal cycler (Invert Logan, Carlsbad, CA, USA).

### 2.5. Bioinformatics and Statistical Analysis

Microbiome bioinformatic analysis was performed using QIIME2 2019.4, with minor modifications, as described in the official tutorial (https://docs.qiime2.org/2019.4/tutorials/, accessed on 18 April 2022). Raw sequence data were demultiplexed using the demux plugin, followed by primer trimming using the cutadapt plugin [27]. Reads were then filtered, denoised, and merged, and chimeric sequences were removed using the DADA2 plugin [28]. We used QIIME2 and the R package (v3.2.0) for sequence data analysis. Alpha diversity indices (Chao1, observed species, Shannon diversity index, Simpson index) at amplicon sequencing variant (ASV) levels were calculated using the ASV table in QIIME2 and visualized using boxplots. Abundance curves were generated at the ASV level to compare the abundance and homogeneity of ASVs in a sample. Beta diversity was analyzed to investigate structural changes in microbial communities between samples and was visualized by principal coordinate analysis (PCoA), multidimensional nonmetric scaling (NMDS), and hierarchical grouping by arithmetic mean methods [29].

Network analysis based on relationships among microbial members is also a common method for microbial community analysis [30]. The underlying purpose of this analysis is to find, through correlation analysis, the intrinsic co-occurrence or co-exclusion patterns in specific microbial communities driven by spatiotemporal changes and environmental processes. Co-occurrence network analysis was performed using SparCC analysis [31] with pseudo-count values between 10 and 6. The cutoff value of the correlation coefficient based on random matrix theory was determined to be 70 using the method implemented in the R package RMThreshold. The network was visualized using the R packages igraph and ggraph. A network hub was considered when the connectivity value within a module (Zi score) was greater than 2.5 and the connectivity value between modules (Pi score) was greater than 0.6 [32]. Microbial functions were predicted using PICRUSt2 (a phylogenetic survey of communities by reconstructing unobserved states) [33], MetaCyc, and KEGG databases.

## 3. Results

### 3.1. Sample Bacterial Sequencing Results

In total, 4,130,615 original sequences were read. After quality control, denoising, and chimera removal, 3,707,271 sequences were still read. Under the action of a nano-aerator, the numbers of high-quality sequences detected after denoising in the water, sediment, and intestinal tract were 89,907 ± 5948, 10,0837 ± 2642, and 156,481 ± 17,409, respectively. Under the action of a common aerator, the numbers of high-quality sequences detected after denoising in the water, sediment, and intestinal tract were 89,555 ± 2709, 96,398 ± 7344, and 135,701 ± 21,330, respectively (Table 1). There were some differences in the quantities of sequences from the water, sediment, and intestinal tract between the two different oxygenator conditions. This difference was most pronounced in the intestinal samples. Compared to that with ordinary aerators, the nano-aerators increased the number of ASVs that could be identified in the water, sediment, and intestinal tract to values of 128, 131, and 58, respectively (Table S1).

### 3.2. Alpha Diversity

**Figure 1:**
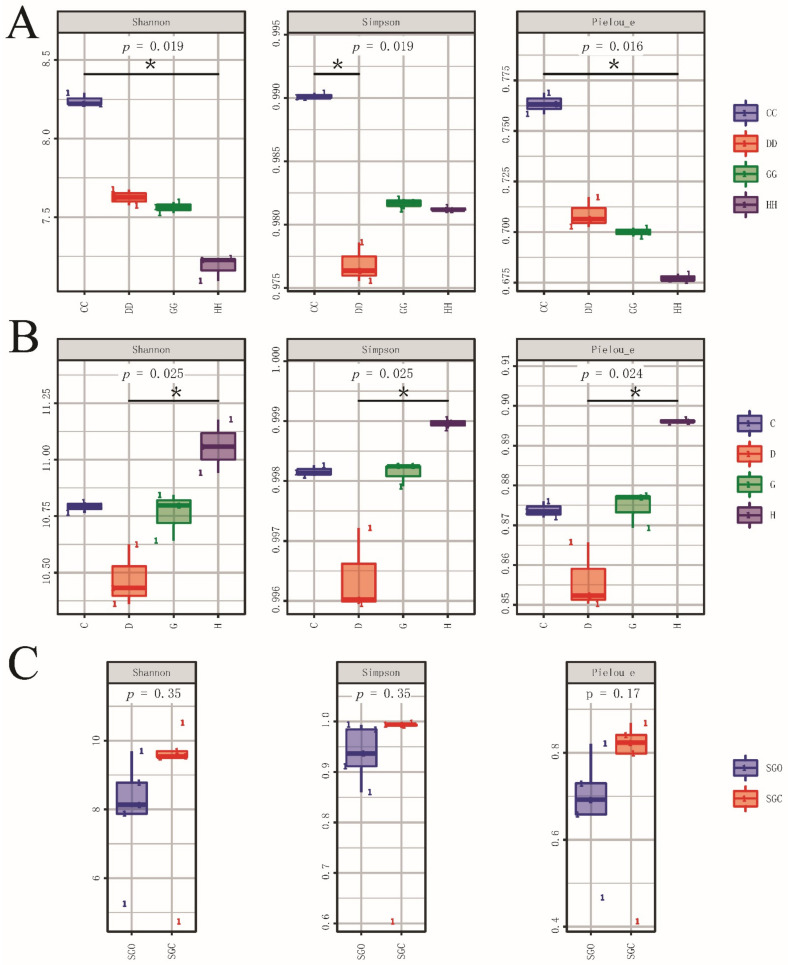
Alpha diversity indices (Shannon, Simpson, Pielou_e) in shrimp farm samples with different oxygenator conditions. Boxplots depict the medians (central horizontal lines), inter-quartile ranges (boxes), and 95% confidence intervals (whiskers). *p*-values are based on the Kruskal–Wallis test. Asterisks indicate statistically significant differences between pairs of values (* *p* < 0.05). (**A**) Bacterial diversity index in water. (**B**) Bacterial diversity index in sediment. (**C**) Diversity index of gut bacteria.

According to Figure 1a, the Shannon, Simpson, and Pielou_e indices of water samples in nano-aerator group (CC) were significantly higher than those in the control group (DD) on 8 August (*P* < 0.05). However, no significant difference was observed in the Simpson index between the treatment group (GG) and control group (HH) on 2 October. In sediment samples, the trends in these indices were approximately the same (Figure 1b). Under short-term oxygenation conditions (8 August), all indices in the treatment group (C) were higher than those in the control group (D). However, with an increase in the test time (2 October), the biodiversity in the treatment group (G) was almost unchanged compared with that in the treatment group (C), and the biodiversity in the control group (H) increased significantly and was higher than that in treatment group (G). There was no significant difference in the alpha diversity index of shrimp gut bacteria between the two groups of samples (Figure 1c).

### 3.3. Abundance Analysis

**Figure 2:**
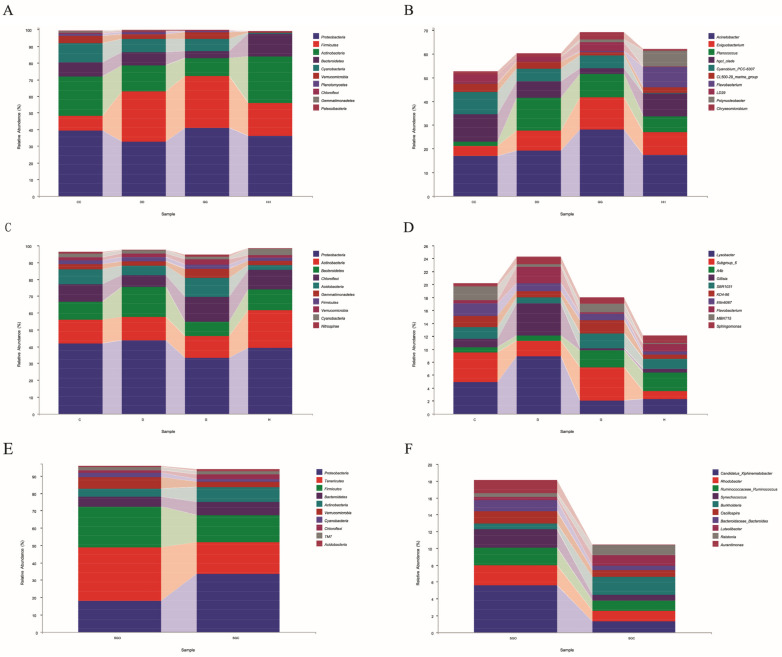
Relative abundances of microbial communities in shrimp farm samples with different oxygenator conditions. (**A**) Phylum level in water. (**B**) Genera level in water. (**C**) Phylum level in sediment. (**D**) Genera level in sediment. (**E**) Phylum level in shrimp gut. (**F**) Genera level in shrimp gut.

At the phylum level, six total main phyla (>1%) were found in the water body of the *L. vannamei* culture pond under different aerator conditions, namely *Proteobacteria*, *Firmicutes*, *Actinobacteria*, *Bacteroidetes*, *Cyanobacteria*, and *Verrucomicrobia*, and the total relative abundance of these dominant phyla accounted for >95% of the entire bacterial flora (Figure 2a). The relative abundance of Proteobacteria among these dominant bacterial phyla was the highest in the CC group and the GG group, accounting for 39.34% and 40.87%, respectively, and indicating that the nanobubble aerator significantly increased the proportion of Proteobacteria in the water body. At the genus level (Figure 2b), the relative abundance of *Subgroup_6* was highest in groups C and G, at 4.54% and 5.12%, respectively. The relative abundance of *Subgroup_6* increased by 0.58% after 4 months of nanobubble aerator treatment, and the relative abundance of *Subgroup_6* decreased by 1.2% after 4 months of turbo-aerator treatment.

In total, nine main phyla (>1%) were found in the sediments from the ponds of *L. vannamei* under different aerator conditions, namely *Proteobacteria*, *Actinobacteria*, *Bacteroidetes*, *Chloroflexi*, *Acidobacteria*, *Gemmatimonadetes*, *Firmicutes*, and *Cyanobacteria* (Figure 2c). Among these phyla, the relative abundance of Proteobacteria was the highest in the 8 August sample, but decreased in the 2 October sample (Figure 2d). The abundance of *Acinetobacter* and *Planococcus* increased by 11.06% and 8.14%, respectively, after 4 months of nano-aerator treatment. However, after 4 months of turbo-aerator treatment, these abundances decreased by 1.83% and 7.16%, respectively.

According to Figure 2e, 10 total major phyla of bacteria (>1%) were found among the gut bacteria of *L. vannamei* under different aeration conditions. The relative abundance of Proteobacteria was twice as high in the control group (SGC) as in the nano-aerator group (SGO), indicating that the nanobubble aerator significantly decreased the proportion of Proteobacteria in the shrimp gut. In contrast, the relative abundance of Tenericutes and Firmicutes in the SGO was twice that in the SGC. At the genus level, the abundance ratios of *Candidatus_Xiphinematobacter*, *Rhodobacter*, *Ruminococcaceae_Ruminococcus*, *Synechococcus*, *Oscillospira, Bacteroidaceae_Bacteroides*, and *Aurantimonas* were higher in the SGO than in the SGC.

### 3.4. Taxonomic Differences in the Microbial Community and Biomarkers

**Figure 3:**
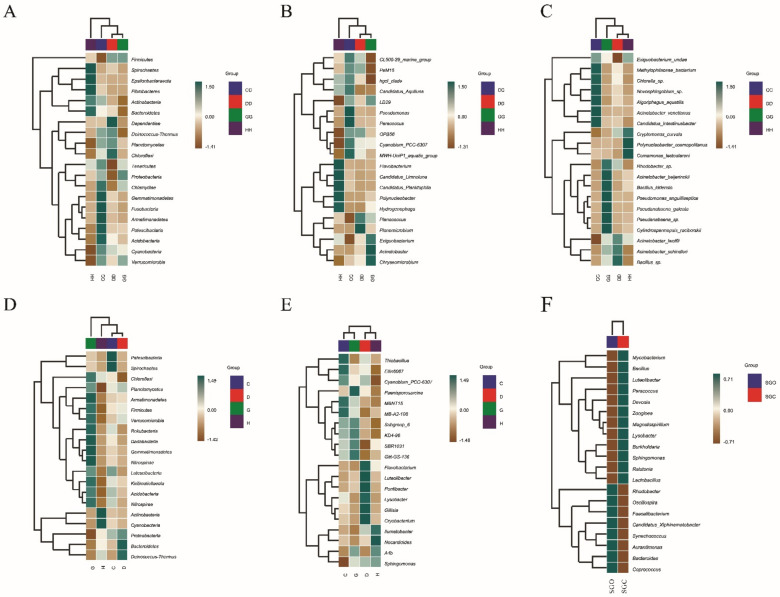
Correlation analysis of microbial communities in shrimp farm samples with different oxygenator conditions. (**A**) Phylum level in water. (**B**) Genera level in water. (**C**) Species level in water. (**D**) Phylum level in sediment. (**E**) Genera level in sediment.

Based on microbiota detection in water samples, we determined that the activity of Spirochaetes at the class level was inhibited in the treatment group (GG) under the influence of long-term oxygenation via the nano-oxygenator (Figure 3a). At the genus level, activity of the beneficial aerobic bacteria *Exiguobacterium* and *Acinetobacter* (Figure 3b) was not only promoted, but activity of the harmful bacterial genus *Flavobacterium* was also inhibited. Another important finding was that under short-term oxygenation conditions, in the treatment group (CC), the activity of one beneficial bacterium, *Chlorella_sp*., and one pathogenic bacterium, *Acinetobacter venetianus*, was increased. However, with an increase in the test time, *A. venetianus* abundance was gradually reduced, and its abundance in the treatment group (GG) was at the lowest level (Figure 3c).

In the four groups of extracted sediment samples, at the class level, Planctomycetes, with an antibacterial effect, was found to have the highest abundance in the treatment group (G) (Figure 3d). One interesting finding was that at the genus level, the activity of a probiotic, *Thiobacillus*, was enhanced in the treatment group (C) under short-term oxygenation conditions. However, with an extension of the test time, the activity of this beneficial bacteria was inhibited in the treatment group (G) (Figure 3e). According to Figure 3f, the level of *Mycobacterium* in the shrimp gut was significantly decreased, but levels of *Rhodobacter*, *Oscillospira*, and *Faecalibacterium* were significantly increased by nano-aerator treatment.

### 3.5. β-Diversity Reveals the Effects of Different Aerators on Microbial Communities

**Figure 4:**
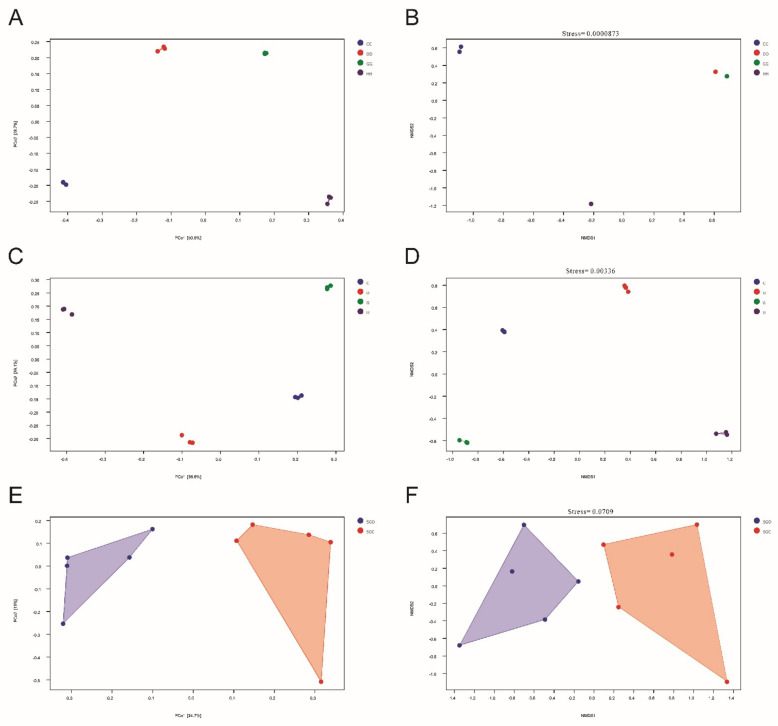
Principal coordinate analysis (PCoA) and nonmetric multidimensional scaling (NMDS) of microbial communities in shrimp farm samples with different oxygenator conditions. (**A**) PCoA of water. (**B**) NMDS of water. (**C**) PCoA of sediment. (**D**) NMDS of sediment. (**E**) PCoA of shrimp gut. (**F**) NMDS of shrimp gut.

The β-diversity index (Bray–Curtis Distance) was obtained using PCoA and NMDS (Figure 4a–f), and there were significant differences in microbial communities between the samples obtained using ordinary and nano-oxygenators. In contrast, intra-group differences were low. These results suggest that the nano-aerators affected the water body and sediment to a certain extent, as well as the composition of bacterial communities in the gut of *L. vannamei*.

### 3.6. Keystone Species Based on Network Analysis

**Figure 5:**
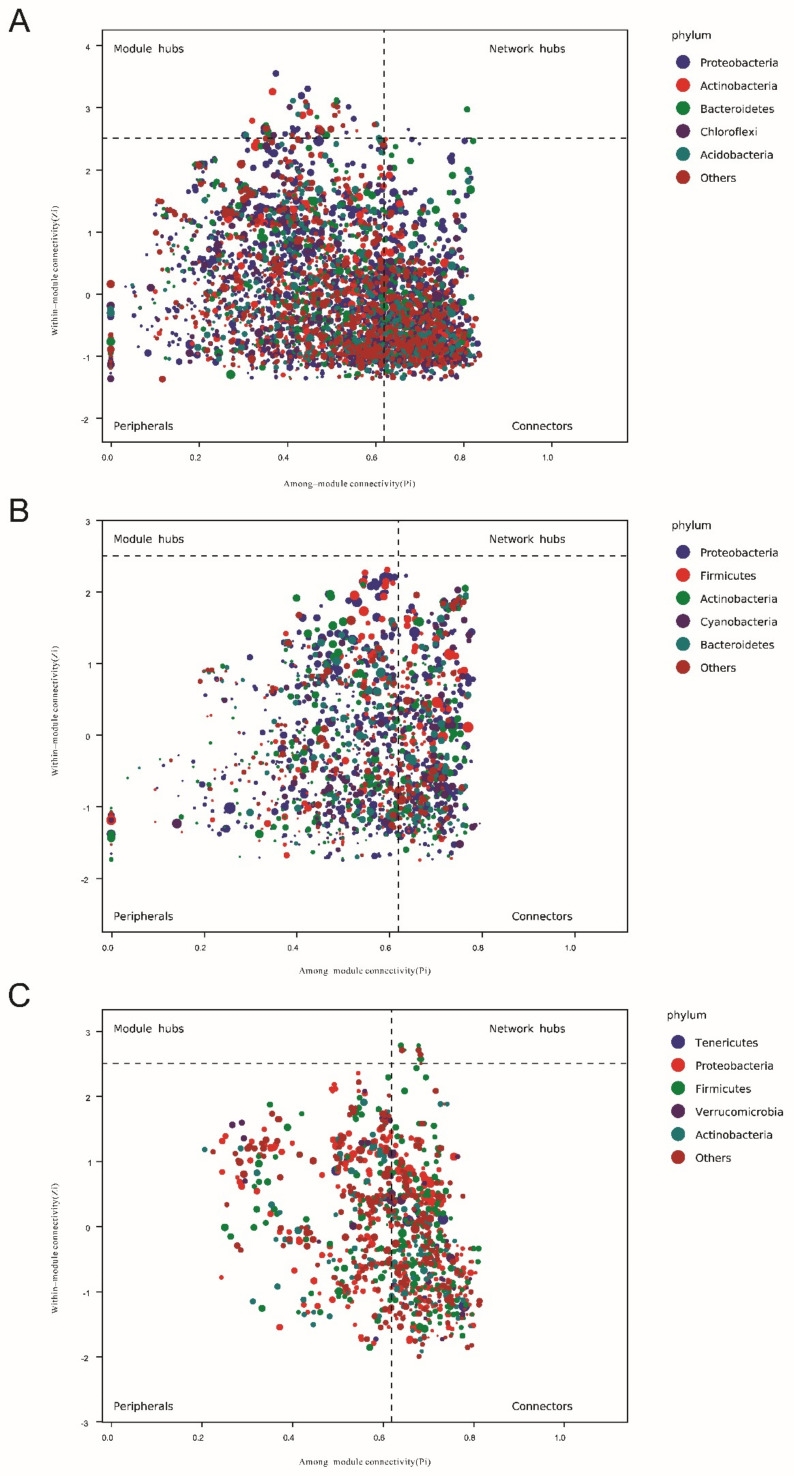
Network analysis of microbial communities in shrimp farm samples with different oxygenator conditions at the phylum level. (**A**) Water. (**B**) Sediment. (**C**) Shrimp gut. Zi: module connectivity, Pi: module connectivity.

A network analysis of the water and sediment microbiota revealed that Bacteroidetes and Proteobacteria are close to generalists (Figure 5a,b), suggesting that changes in the proportions of these two phyla have the potential to affect the composition of the microbiome throughout the culture environment. In addition, our network analysis revealed that Firmicutes comprised the central community (Figure 5c). This suggests that changes in the ratio of Firmicutes are sufficient to influence the composition of the entire gut microbiome.

### 3.7. Potential Function of the Gut Bacterial Community

**Figure 6:**
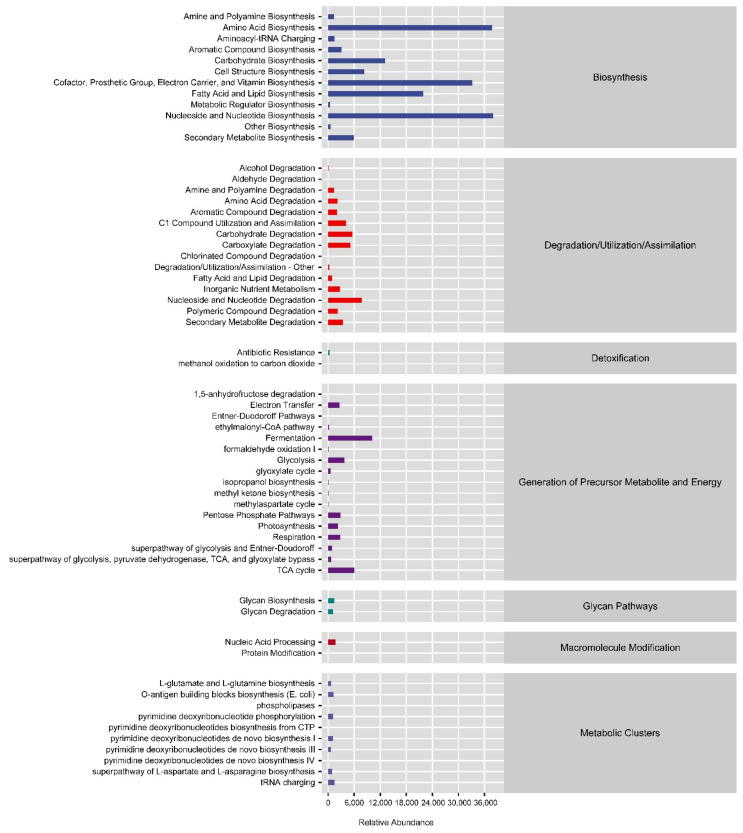
Relative abundances of metabolic pathways in shrimp farm samples different oxygenator conditions. Blue: Biosynthesis; Red: Degradation/Utilization/Assimilation; Light green: Detoxification; Modena: Generation of precursor metabolite and energy; Olive green: Glycan pathways; Deep red: Macromolecule modification; Orchid: Metabolic clusters.

We next determined the abundances of metabolic pathways active in the microbiome by consulting various metabolic pathway databases and applying calculation methods (Figure 6). The biosynthesis pathway category was associated with the most enriched pathways, including amino acid biosynthesis; cofactor, prosthetic group, electron carrier, and vitamin biosynthesis; fatty acid and lipid biosynthesis; and nucleoside and nucleotide biosynthesis.

## 4. Discussion

The use of aerators not only increases the dissolved oxygen content in the water, but also fully inhibits the growth of anaerobic bacteria, preventing the deterioration of the pool water and threatening the living environment of aquatic organisms [34]. Currently, most of the commonly used aerators have limitations, such as a high laying cost, a fixed aeration area, and high energy consumption [4]. In contrast, the micro- and nanobubbles released by the nano-oxygenator will not increase and do not appear to float, and the oxygen-increase efficiency is higher [35]. Yao et al. [36] used nano-aeration equipment to conduct the biological denitrification treatment of aquaculture wastewater. In this study, the effects of a nano-aerator and turbo-aerator on the microflora of a water body and bottom mud over 4 months were compared. A nano-aerator was selected because in recent years, nanobubble technology has attracted much attention, and compared with an ordinary aerator, a nano-aerator exhibits lower power consumption and an obvious energy-saving effect [37].

To the best of our knowledge, this study is the first to describe the effects of nano-aerators on the microbial community structure of water and sediment in a pond of *L. vannamei*. Our research results showed that the application of nano-aerators changes the structures of water and sediment microbial communities to a certain extent compared to those with ordinary aerators. The results of α-diversity analysis showed that there were significant differences in the composition of microbial communities in the water body and sediment. Simpson, Shannon, and Pielou_e indices showed that nano-aerators (groups CC and GG) increased the diversity, richness, and homogeneity of microbial communities in the water body. The effect of nano-aerators on the microbial community of sediments could be time-sensitive. Under short-term aeration conditions, the microbial diversity in the CC group was higher than that in the DD group; however, with an increase in the aeration time, the microbial diversity in the sediments of the HH group exceeded that in the GG group, as indicated by the results of the rarefaction curve and the abundance rank curve (Figure S1 and S2). According to the results of the hierarchical clustering heat map analysis, activities of the beneficial bacteria genera *Exiguobacterium* and *Acinetobacter* and beneficial bacterium *Chlorella_sp* in the water body all increased owing to oxygenation mediated by the nano-aerator. The abundance of Spirochaetes, the causative agent of oyster disease, decreases in water bodies under the influence of nano-aerators [38]. During one experiment, the abundance of the pathogenic bacterium *A. venetianus* [39] first increased and then decreased. Studies have shown that the use of *Acinetobacter KU011TH* as a probiotic for juvenile catfish significantly improves lysozyme activity and respiratory activity in the body [40]. Some species of *Exiguobacterium* are gram-variable, rod-shaped organisms capable of producing extracellular enzymatically active substances to break down chemical structures to remove organic compounds from aquaculture waste [41]. Ekasari et al. [42] demonstrated that the addition of *Chlorella_sp.* improves the physical and biochemical properties of bioflocs and the growth of *Macrobrachium rosenbergii* cultured in biofloc systems under laboratory conditions. In the sediment samples, Planctomycetes, with antibacterial effects, was most abundant in the treatment group (G). A previous study [43] assessed the antibiotic susceptibility profile of six phylogenetically distinct Planctomycetes strains and found that all exhibited resistance to β-lactams, aminoglycosides, and glycopeptides. In this experiment, the abundance of *Thiobacillus* [44] in the treatment group gradually decreased, which might be because the nano-aerator cannot fully stir the bottom mud and cannot increase the dissolved oxygen content in the bottom water well, resulting in the decreased abundance of *Thiobacillus*.

The results of the alpha diversity analysis showed that the overall richness and diversity of gut microbiota were not significantly different between the two groups of samples. However, we found that the species composition distribution of the gut microbiota was very peculiar. In the analysis of the species composition, it was found that the abundance of various dominant genera in the treatment group (SGO) was significantly higher than that in the control group (SGC). According to the results of hierarchical clustering heat map analysis, the activities of beneficial bacteria in the gut, *Rhodobacter*, *Oscillospira*, and *Faecalibacterium*, were all increased owing to the effect of the nano-aerator. Previous studies [45] have demonstrated that feeding *L. vannamei* with *Rhodobacter sphaeroides* protein instead of fish meal can improve its growth performance, maintain gut health, and enhance antioxidant capacity and immunity. In this experiment, *Oscillospira* and *Faecalibacterium*, of which levels were promoted by the oxygen-enhancing effect of the nano-aerator, were found to have certain effects on human health and immunity. *Oscillospira* exerts positive regulatory effects directly or indirectly on aspects related to obesity and chronic inflammation [46]. *Faecalibacterium prausnitzii* inhibits the growth of breast cancer cells by inhibiting the IL-6/STAT3 pathway [47]. We believe that consumers can increase the content of the two aforementioned bacterial taxa in the intestinal tract by ingesting *L. vannamei* subjected to growth conditions based on oxygenation with the nano-aerator, thereby reducing some intestinal inflammation and the incidence of breast cancer. Moreover, this could reduce the abundance of harmful bacteria in the gut, such as *Mycobacterium*. Davidovich et al. were the first to describe the process through which mycobacteria, as pathogenic bacteria, infect red crayfish [48].

Nano-oxygenators can use many gas media, such as hydrogen (H_2_), nitrogen (N_2_), and ozone (O_3_). However, existing studies have shown that nano-oxygenators are usually used in conjunction with ozone to kill pathogenic bacteria in cultured water. Jhunkeaw et al. [49] demonstrated similar disinfection efficiency with ozone nanobubble treatment against pathogenic bacteria in freshwater for the first time. In this experiment, the potential pathogen that causes *L. vannamei* red-leg disease in freshwater aquaculture was also effectively reduced when the nano-aerator used air as the gas medium. Therefore, we speculate that the nano-aerator can also kill some pathogenic bacteria in the water body and sediment when using the air medium. In this experiment, the nano-oxygenator effectively inhibited the activity of Bacteroidetes, Spirochaetes, and other anaerobic bacteria in water. This is similar to the previous results of Trong et al. [50], who demonstrated that bubbles reduce the biomass through hydrophobicity, thereby inhibiting bacterial growth. This proves that the nano-oxygenator can also inhibit anaerobic bacteria when using air as the gas medium. We believe that this is an important finding for the future practical production and selection of aeration equipment.

Although this study revealed some important findings, there were also some limitations, and the results of high-throughput sequencing could also be affected by various factors, such as individual sampling methods and experimental methods. Owing to the small sample size, the aforementioned influencing factors cannot be excluded. However, the results are still helpful to understand the structure of the microbial ecosystems associated with species and provide a theoretical basis for actual production. Therefore, experimenting with nano-aerators in different farming modes will be of great importance for future research. In addition, few studies have been conducted on the effects of nano-aerators on the crustacean gut microbiota in culture ponds. In conclusion, our findings improve the understanding of the microbial community structure in water and sediments of aquaculture ponds using micro-nano-aerators. These findings could contribute to improved farming environments to enhance the quality and health of crustaceans in aquaculture environments.

## 5. Conclusions

We succeeded in discovering changes in the pond water, sediment, and shrimp gut microbial communities of *L. vannamei*. Our results showed that the microbial community diversity in water decreases gradually with an increase in the experimental time, but the biological diversity in the treatment group was always higher than that in the control group owing to the effect of oxygen addition via the nano-oxygenator. This could be because the nano-oxygenator has better oxygen-increasing effects, and when the nanobubble bursts, it produces a large number of hydroxyl radicals, which degrade some pollutants in the water, thus purifying the water and providing a more suitable environment for various microorganisms in the water. In addition, according to the cluster analysis heat map, the nano-oxygenator even affected the species composition of the microbial community in the aquaculture pond water, bottom sediment, and shrimp gut to a certain extent, and increased the proportion of some probiotics. In view of the results obtained, our study and subsequent studies based on our results might prove that nano-aerators are useful for improving the aquaculture of *L. vannamei*. However, different conditions such as temperature, salt concentration, or breeding density could impact results, and more experiments still need to focus on those environment factors.

## Figures and Tables

**Table 1:** Sequence after denoising in water, sediment, and shrimp intestinal samples under the action of nano-aerator and control.

	Water		Sediment		Shrimp Intestine	
	Nano-Aerator	Control	Nano-Aerator	Control	Nano-Aerator	Control
Total sequences	98,576 ± 6462	96,102 ± 2841	116,117 ± 2919	111,931 ± 7987	170,603 ± 18,535	148,246 ± 22,084
Sequences after denoising	89,907 ± 5948	89,555 ± 2709	100,837 ± 2642	96,398 ± 7344	156,481 ± 17409	135,701 ± 21,330

## Data Availability

The raw reads have been deposited into the NCBI database (BioSample accession numbers SAMN27484803, SAMN27484804, and SAMN27484805).

## Supplementary Materials

The following supporting information can be downloaded at: https://www.mdpi.com/article/10.3390/microorganisms10071302/s1, Figure S1: (A) abundance grading curve of water microbial community, (B) sparse curves of water microbial communities (the exponent type is Shannon), (C) sparse curves of water microbial communities (the exponent type is Pielou_e); Figure S2: (A) abundance grading curve of sediment microbial community, (B) sparse curves of sediment microbial communities (the exponent type is Shannon), (C) sparse curves of sediment microbial communities (the exponent type is Pielou_e); Table S1: the average number of detectable ASVs species in water, sediment, and intestinal samples under the action of nano-aerator and control.
