# Supplementary material for: Effects of Nano-Aerators on Microbial Communities and Functions in the Water, Sediment, and Shrimp Intestine in Litopenaeus vannamei Aquaculture Ponds
Source: Microorganisms. 2022 Jun 27;10(7):1302. doi: 10.3390/microorganisms10071302 (PMC9317398; doi:10.3390/microorganisms10071302)
Supplement: Supplementary file 1 [file microorganisms-10-01302-s001.zip › Table S1.pdf]

Table S1 The average number of detectable ASVs species in water, sediment, and intestinal samples under the action of nano-aerator and control.

	Water		Sediment		Shrimp intestine	
	Nano-aerator	Control	Nano-aerator	Control	Nano-aerator	Control
A total number of ASVs	1784±86	1656±125	5136±163	5005±369	3519±618	3461±655
