# Supplementary figures and images for: Effects of Nano-Aerators on Microbial Communities and Functions in the Water, Sediment, and Shrimp Intestine in Litopenaeus vannamei Aquaculture Ponds
Source: Microorganisms. 2022 Jun 27;10(7):1302. doi: 10.3390/microorganisms10071302 (PMC9317398; doi:10.3390/microorganisms10071302)

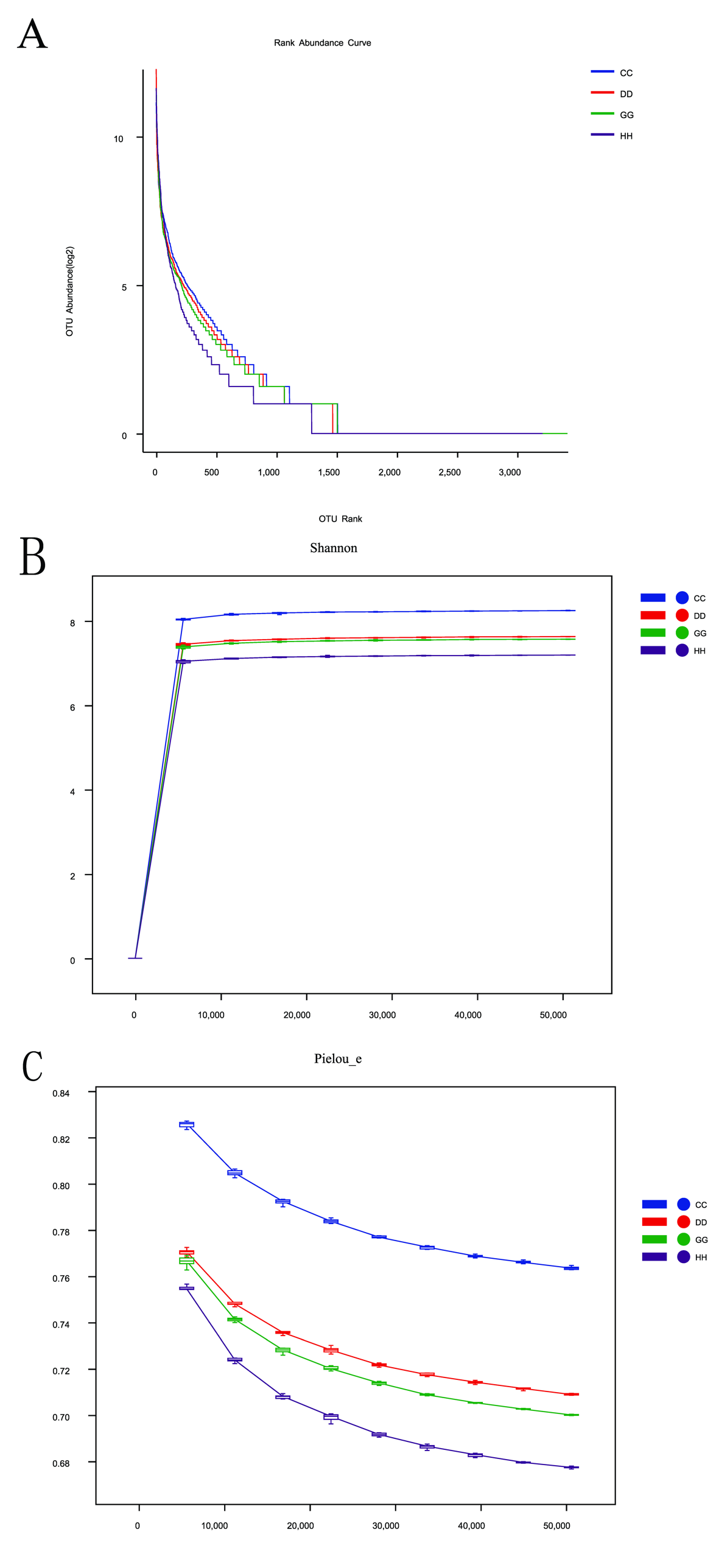

Supplement: Supplementary file 1 [file microorganisms-10-01302-s001.zip › Figure S1.jpg]

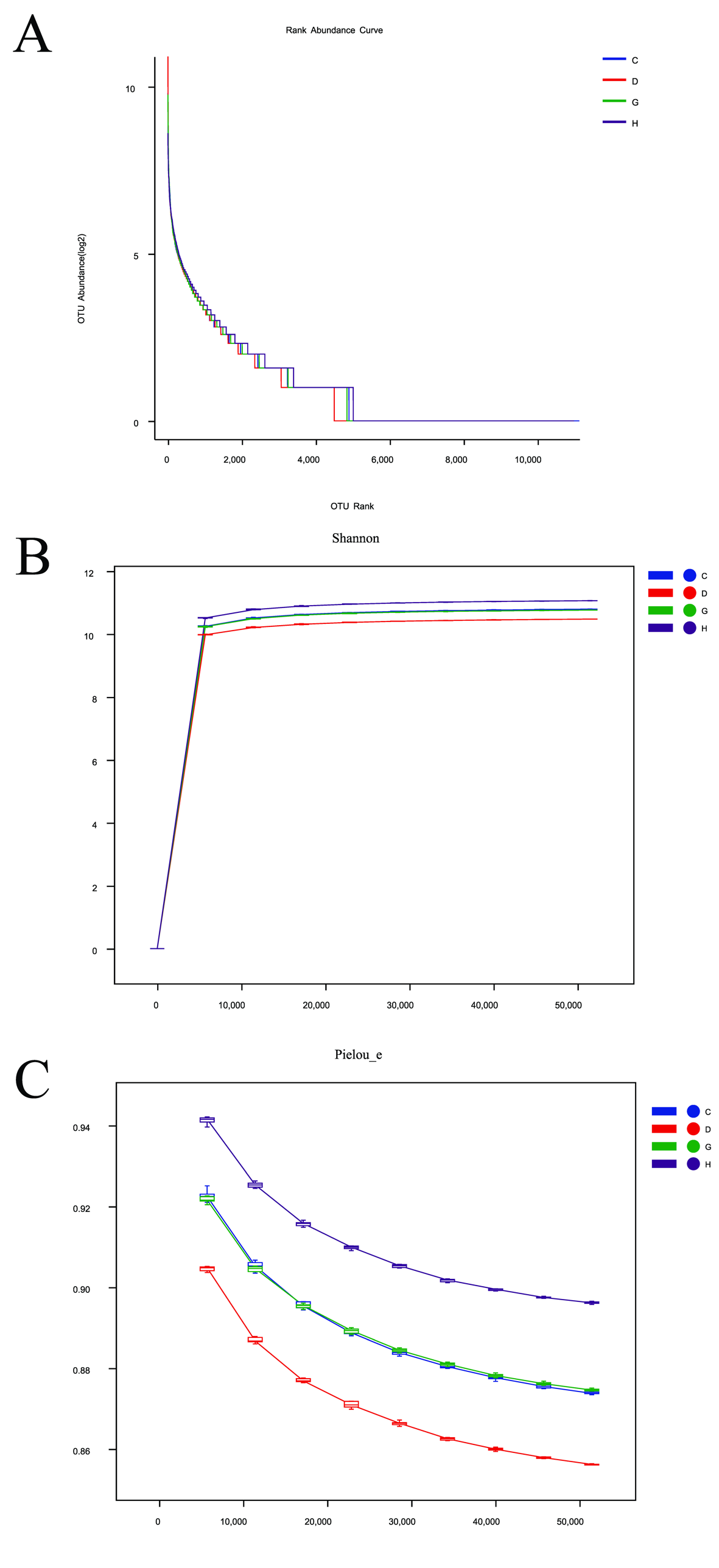

Supplement: Supplementary file 1 [file microorganisms-10-01302-s001.zip › Figure S2.jpg]
